# Rescuing gait under cognitive load: the benefits of Tai Chi for MCI

**DOI:** 10.3389/fphys.2026.1770084

**Published:** 2026-03-23

**Authors:** Cenyi Wang, Houyu Liu, Xiang Gu, Jingzhi Han, Bingqing Wang, Guodong Wang

**Affiliations:** School of Physical Education and Sports Science, Soochow University, Suzhou, China

**Keywords:** dual task, gait, mild cognitive impairment, old adults, Tai Chi

## Abstract

**Introduction:**

Tai Chi has been shown to exert beneficial effects on cognitive function in older adults; however, the mechanisms underlying its improvement of postural control and mobility remain unclear. This study aimed to investigate the effects of a 3-month Tai Chi training intervention on cognitive function and postural control in older adults, thereby providing a scientific basis for exercise recommendations for this population.

**Method:**

This study aimed to evaluate the effects of 8 forms of Tai Chi training over a period of 3 months on gait characteristics. A total of 34 older adults (25 females) completed the full study protocol. Walking kinematic data were collected using the MyoMotion 3D motion analysis system under single-task and three dual-task conditions (walking while counting, carrying water, and naming). Spatiotemporal parameters, including step length, cadence, and lower limb joint angles, were extracted through gait cycle analysis. The control group maintained their habitual daily activities. Repeated measures analysis of variance (ANOVA) was used to compare differences between groups and across time points (pre- and post-intervention), with the significance level set at 0.05.

**Result:**

Baseline characteristics were comparable between groups (p >0.05). Post-intervention, the experimental group demonstrated significant improvements in gait spatial parameters, characterized by increased normalized step and stride lengths and decreased step width compared to controls (p <0.05). Temporally, the experimental group exhibited an increased percentage of stance phase. Kinematically, the intervention significantly enhanced hip range of motion (p <0.01) and altered knee and ankle joint angles at specific gait events. Notably, a significant group-by-task interaction was observed for maximum hip extension (p <0.05), indicating that Tai Chi training differentially optimized hip mechanics across varying dual-task conditions. Additionally, the experimental group showed significantly improved overall cognitive function (p < 0.01).

**Conclusion:**

The Tai Chi intervention effectively improved gait stability, balance, and overall gait performance under dual-task conditions in older adults with MCI, thereby reducing the risk of falls. These findings support Tai Chi as a safe, cost-effective, and scalable non-pharmacological intervention for fall prevention in this population.

## 1 Introduction

In recent years, population aging has emerged as a significant global challenge. In 2023, the population of individuals aged 65 and older in China exceeded 200 million. This figure is projected to reach 480 million by 2050, accounting for 37.8% of the total population (The L, 2022). Consequently, the safety and quality of life of the elderly population have garnered increasing attention from researchers (National Bureau of Statistics of China, 2023).

Notably, cognitive decline remains a primary focus of health concerns within the elderly population. Cognition is defined as the brain’s capacity for information processing, storage, and retrieval, manifesting primarily in domains such as executive function, attention, and memory. However, cognitive deterioration is often accompanied by a concomitant decline in motor function, thereby precipitating the occurrence of falls. Relevant studies indicate that the mortality risk for older adults with cognitive impairment is 2 to 3.5 times higher than that of their cognitively intact peers. Furthermore, the severity of cognitive impairment is positively correlated with mortality risk (Agüero-Torres et al., 1999). With the aging population, the prevalence of cognitive impairment is continuously rising. As of 2020, the number of individuals aged 60 and older in China living with dementia reached 15.07 million, while those with mild cognitive impairment (MCI) totaled a staggering 38.77 million (Jia et al., 2020; Stevenson-Hoare et al., 2023). Moreover, the incidence of MCI increases progressively with age. The prevalence among Chinese adults aged 65 and older has reached 20.8%, with approximately 6% to 10% of MCI patients progressing to dementia (Ding et al., 2015; Bora and Yener, 2017; Anderson, 2019). However, given the current lack of effective curative treatments for dementia, the MCI stage is internationally recognized as the optimal window for preventing Alzheimer’s disease (AD) and other forms of dementia. Therefore, intervention during the MCI stage is of paramount importance (Li et al., 2023).

Common interventions for patients with MCI can be broadly categorized into five modalities: pharmacological, cognitive, physical exercise, dietary, and traditional Chinese medicine (TCM) non-pharmacological interventions (Wang et al., 2023). Among these, physical exercise interventions can be further stratified into aerobic, resistance, mind-body, and multi-modal exercises. Tai Chi, a mind-body exercise characterized by the integration of postural control, torso rotation, weight shifting, and strength enhancement, is widely favored by the elderly for its gentle, slow movements and meditative focus (Hong and Li, 2007; Huang et al., 2022). A substantial body of research demonstrates that Tai Chi can effectively improve global cognitive function in older adults(13,14,15). Li et al. (2014) assessed the global cognitive ability of elderly patients with MCI following 14 weeks of Tai Chi training using the Mini-Mental State Examination (MMSE) and observed significant improvements. Similarly, Chen et al. (2023) reported a significant increase in cognitive scores following Tai Chi intervention. In a comparative study involving adults aged 60 to 70, Zhang et al. (2014) evaluated the effects of swimming, Tai Chi, and square dancing. They found that Tai Chi yielded the most significant cognitive benefits, proving superior to the other two forms of aerobic exercise. Furthermore, Tai Chi exerts a significant positive impact on motor function in elderly individuals. It specifically enhances muscle strength, balance, and postural control, thereby reducing the risk of falls (Wolfson et al., 1996; Wu, 2002).For the elderly, aging is inevitably accompanied by a decline in physical function; consequently, long-term high-intensity exercise training often exceeds their physiological tolerance. In contrast, Tai Chi—a traditional Chinese fitness practice—serves as a low-to-moderate intensity aerobic exercise. It requires mental tranquility and physical relaxation, integrating intent, movement, and abdominal breathing, making it particularly well suited for the elderly population (Hong and Li, 2007; Li et al., 2023). Specifically, 8-form Tai Chi incorporates the eight most fundamental movements of Yang-style large-frame Tai Chi. Designed with the age and physical characteristics of older adults in mind, this version eliminates high-load maneuvers such as breath-holding and sudden exertion of force, thereby avoiding excessive burden on the respiratory and cardiovascular systems. Characterized by its concise content and simple forms, it is easy to learn and memorize. Accordingly, it has been listed as a recommended exercise in the Chinese Expert Consensus on Rehabilitation Management of Alzheimer’s Disease (2019) (The Neurodegenerative Disease Committee of the Chinese Microcirculation Society, 2020) and is deemed highly suitable for elderly patients with MCI (Li et al., 2014).

To date, the majority of research regarding the efficacy of Tai Chi interventions in elderly patients with MCI has remained concentrated within the cognitive domain. Few scholars have investigated its effects from the perspective of postural control and mobility. Specifically, there is a relative scarcity of studies examining the influence of Tai Chi on gait postural control in this population, particularly under dual-task conditions. Therefore, the present study aims to investigate the impact of an 8-form Tai Chi intervention on gait postural control under dual-task conditions in elderly patients with MCI. The findings intend to provide a theoretical basis and practical guidance for enhancing postural control and reducing the risk of falls in the elderly MCI population.

The hypotheses of this study were as follows: (1) The 8-form Tai Chi intervention would enhance gait postural control in elderly patients with MCI; and (2) Following the intervention, the changes in gait postural control would differ between motor-cognitive and motor-motor dual-task conditions or among distinct cognitive dual-task conditions.

## 2 Materials and methods

### 2.1 Participants

Participants aged 60 years and older with mild cognitive impairment (MCI) were recruited from communities and elderly care institutions in Suzhou, Jiangsu Province. The Montreal Cognitive Assessment (MoCA) was employed to screen the subjects. The MoCA has a maximum total score of 30, with a score of ≥ 26 indicating normal cognitive function (Nasreddine et al., 2005). The sample size was calculated using G*Power software with an effect size of 0.4, a significance level (α) of 0.05, and a statistical power of 0.8. Based on this calculation, a minimum of 34 participants was required. To account for potential attrition, a total of 40 participants were enrolled. Participants were randomly assigned to either the experimental group or the control group using a computer-generated random number sequence (SPSS, 26.0), stratified by age and gender. Allocation concealment was implemented using sequentially numbered, opaque, sealed envelopes prepared by an independent researcher not involved in recruitment or assessment. Outcome assessors and the statistician were blinded to group allocation throughout the study. Baseline characteristics of the participants were collected. This study was approved by the Ethics Committee of Soochow University (Approval No. SZDX20211227H03).

#### 2.1.1 Inclusion criteria

Participants were included if they met the following criteria:(1) Aged 60 years or older; (2) A MoCA score ranging from 18<score<26 (Nasreddine et al., 2005), and capable of participating in the experiment; (3) Both the participants and their family members understood the study, agreed to cooperate with the experimental procedures, and voluntarily signed the informed consent form; (4) Normal physical function, with the ability to complete Tai Chi training and walking activities.

#### 2.1.2 Exclusion criteria

Participants were excluded based on the following criteria: (1) aged < 60 years; (2) presence of severe medical conditions, such as severe cardio-cerebrovascular diseases or advanced malignancies; (3) diagnosis of severe dementia, severe cognitive impairment, other psychiatric disorders, or brain injuries that rendered the participant unable to complete the required assessments; and (4) restricted physical function or physical disabilities that prevented the completion of the exercise intervention and specified movements.

### 2.2 Experimental procedures and methods

**Figure 1:**
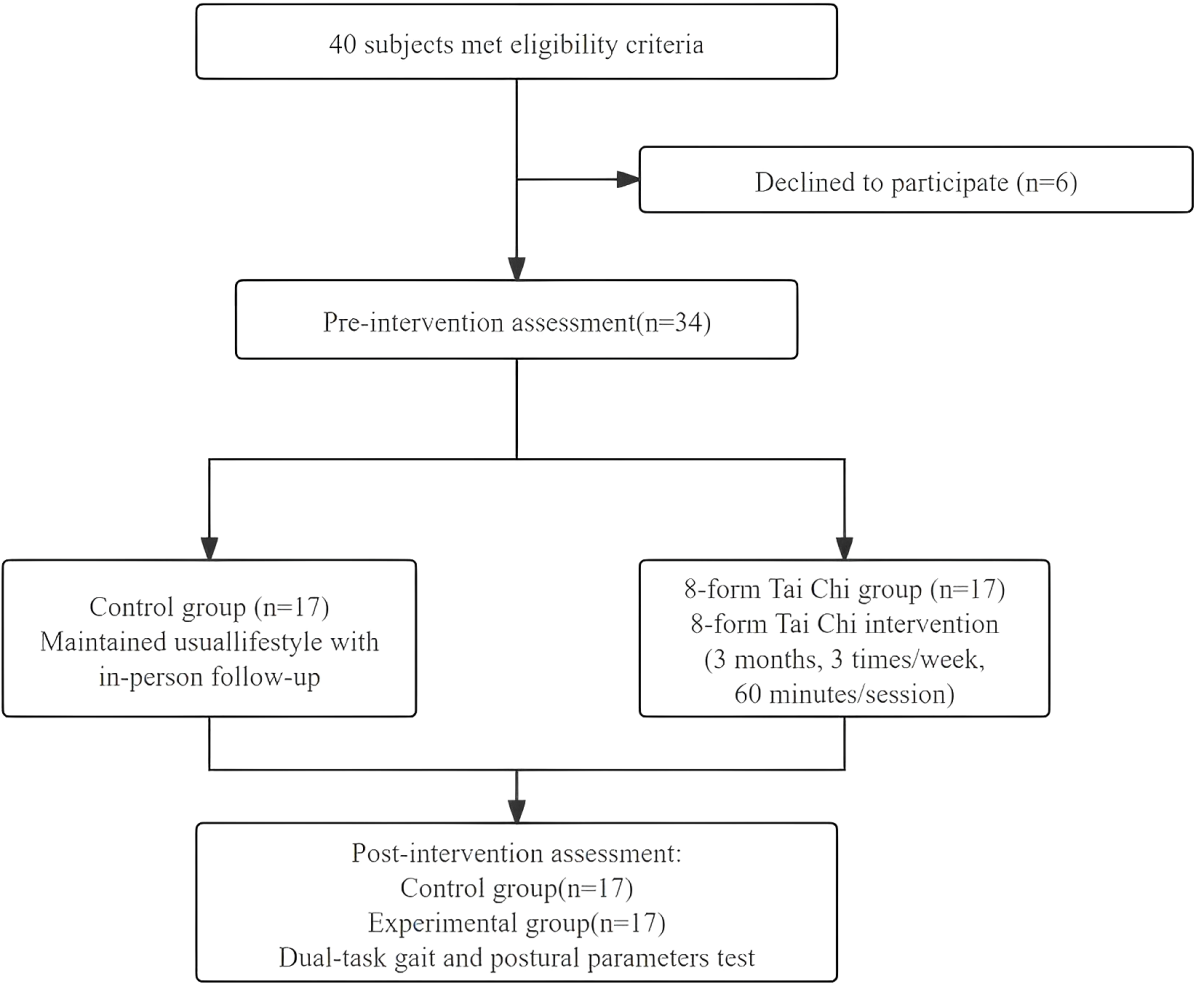
Flow chart of participant inclusion.

A total of 40 elderly patients with MCI were enrolled and assigned to two groups for the intervention. During the study, 6 participants dropped out (1 refused the post-intervention assessment, 2 left the study area (Suzhou), and 3 withdrew due to an inability to persist with the Tai Chi practice). Ultimately, 34 participants completed the study. The detailed experimental procedures and the study flow are illustrated in **Figure 1**.

#### 2.2.1 Gait function test

The MyoMotion 3D motion capture and analysis system (Noraxon, USA) was employed to acquire kinematic characteristics during walking. The system comprises multiple inertial sensors, a wireless signal receiver, and a data acquisition and analysis software module. The MyoMotion system supports the simultaneous operation of up to 36 sensors for collecting kinematic parameters, with a sampling frequency ranging from 100 to 200 Hz. The angular accuracy is ±1° in the sagittal and coronal planes and ±2° in the transverse plane. The maximum measurement range is 2000°/s for angular velocity and 16 g for acceleration. Simultaneously, a standard video camera (Sony, Japan) was used to record the walking process at a sampling rate of 100 Hz. The MyoMotion system and the video camera were synchronized using an external flash trigger. A flash unit connected to the MyoMotion receiver was activated at the start of each trial, and the camera recorded the flash, enabling frame−accurate alignment of the 100 Hz sensor data and the 100 Hz video frames. The recorded videos were analyzed using SIMI Motion software (SIMI Reality Motion Systems, Germany) to obtain gait data.

**Figure 2:**
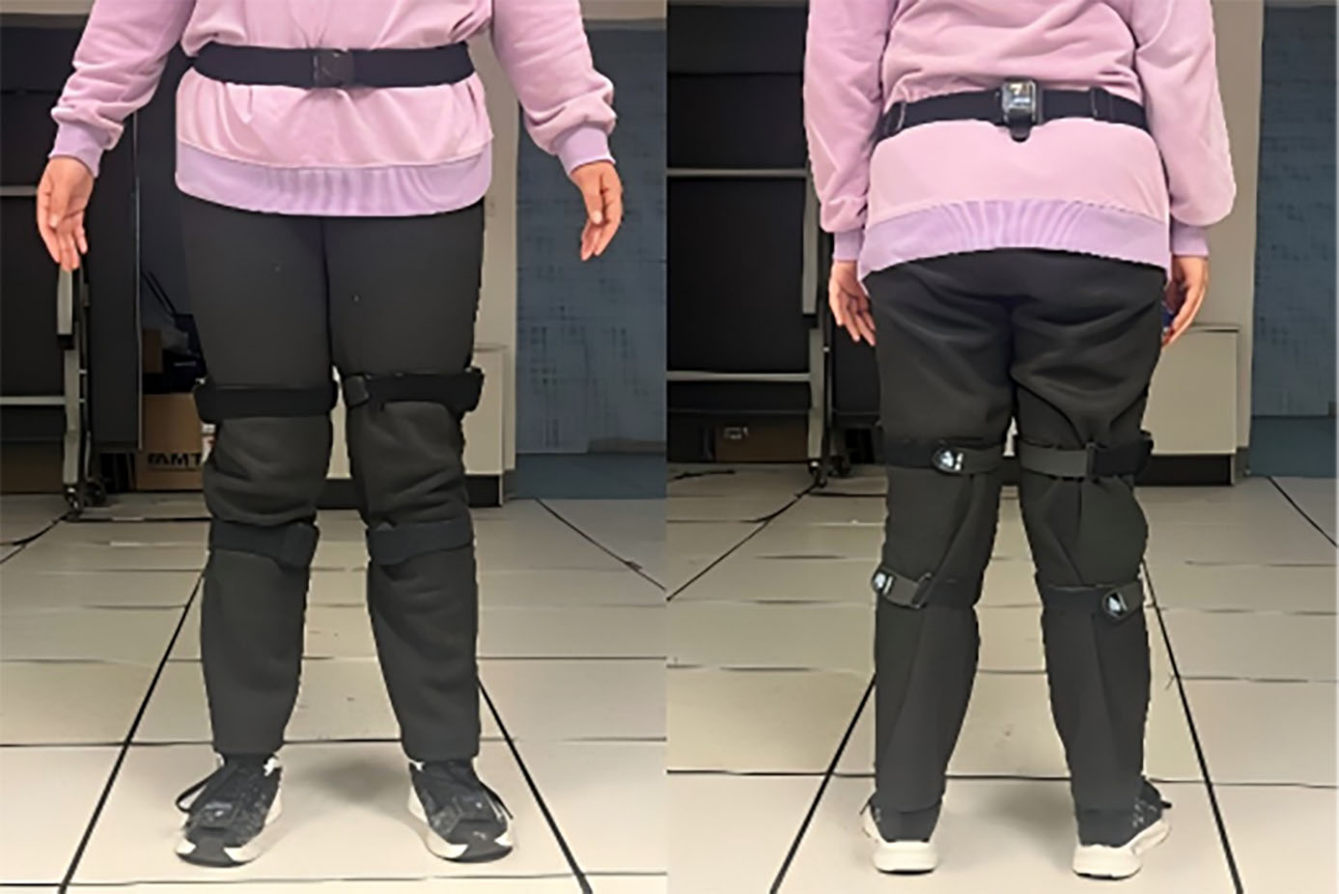
MyoMotion Inertial Sensor Placement Diagram (Left. Sensor Placement, Anterior View. Right. Sensor Placement, Posterior View).

Researchers secured the sensors using straps to the participants’ lumbar region, bilateral thighs, bilateral shanks, and feet. The placement of the sensors is illustrated in **Figure 2**. A sampling frequency of 100 Hz was selected for this study. Prior to testing, participants were instructed to stand still in an upright position for 10 seconds to perform posture calibration and establish a static model. Subsequently, the participants walked freely to acclimatize to the equipment. Once adapted, they were instructed to walk a distance of 10 meters with a natural gait, during which data were collected by the researchers. Data trials were considered valid if the participant exhibited a natural gait without pauses or other deviations from the experimental protocol. A minimum of three valid trials were collected for each participant. The raw sensor data were processed using the proprietary algorithms of the MyoMotion system, which apply a Kalman filter for sensor fusion and orientation estimation. A 10 Hz low−pass filter setting within the system was used for filtering. No additional offline filtering was applied to the kinematic data.

#### 2.2.2 Dual-task types and testing

Participants were required to perform walking tests under dual-task conditions. Three distinct dual-task paradigms were employed: Walking while Counting, Walking while Carrying Water, and Walking while Naming. Walking while Counting: This task required participants to perform a serial subtraction of three while walking naturally. At the start of the walk, the researcher provided a random number between 100 and 300. Participants were instructed to perform the calculations and articulate the process aloud during the walk (e.g., “235 minus 3 equals 232, 232 minus 3 equals 229, 229 minus 3 equals 226…”) (Wang et al., 2024). Walking while Carrying Water: This task required participants to carry a cup of water while walking naturally, with instructions to maintain stability and prevent spillage. Prior to each trial, the water level in the paper cup was checked to ensure that it reached a standardized marker line (Wang et al., 2024). Walking while Naming: This task required participants to perform a verbal fluency task while walking naturally. At the beginning of the walk, the researcher provided a specific category from the following options: vegetables, home appliances, furniture, transportation, or fruits. Participants were required to continuously verbally list items belonging to the assigned category (e.g., upon hearing the category “vegetables,” the participant would list items such as “Chinese cabbage, bok choy, tomato,” etc.) until the walking trial was completed (Plummer-D’Amato et al., 2012).

#### 2.2.3 Selection of indicators

A complete gait cycle was analyzed by extracting data based on characteristic events from the initial heel strike of one foot to the subsequent heel strike of the same foot. The interval from the initial heel strike to toe-off was defined as the stance phase, while the interval from toe-off to the subsequent heel strike was defined as the swing phase. The gait function indicators were categorized into spatial parameters and temporal parameters.

Spatial parameters included step length (SL), stride length (StL), step width (SW), and lower limb joint angles. For the sign conventions used in this study, flexion of the hip and knee joints was defined as positive (+), while extension was defined as negative (-). For the ankle joint, dorsiflexion was defined as positive (+), and plantar flexion was defined as negative (-). The temporal parameters included step frequency (SF), step time (ST), gait cycle duration (GC), stance phase percentage, and swing phase percentage. Additionally, we recorded the accuracy rates(AR), reaction times(RT), and the number of correct naming responses(CNR) for the dual-task of counting and naming.

#### 2.2.4 Intervention protocol

The experimental group underwent a three-month, 8-form Tai Chi training program, with sessions held three times per week, each lasting 60 minutes. The control group was instructed to maintain their habitual daily activities and received bi-weekly follow-ups via telephone or face-to-face visits conducted by the researchers. To ensure consistent testing conditions, a standardized 10-minute warm-up protocol was performed prior to all assessments, including both baseline and post-intervention tests. The warm-up consisted of 5 minutes of walking at a self-selected comfortable pace, followed by 5 minutes of dynamic stretches targeting the lower extremities (hips, knees, and ankles), with each stretching movement repeated 8–10 times. Moreover, for the experimental group, all post-intervention assessments were conducted within 48 to 72 hours after the final Tai Chi session. This time window was selected to preserve the cumulative training adaptations while excluding potential acute effects of the last exercise session.

**Figure 3:**
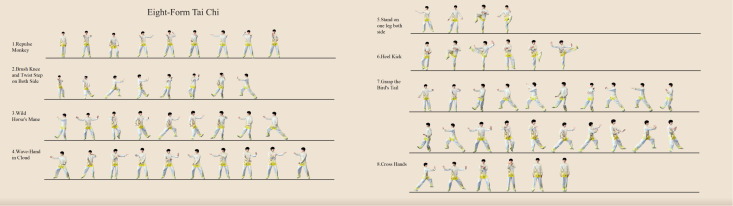
Eight-Form Tai Chi.

The intervention adopted the Eight-Form Tai Chi, developed by the Oregon Research Institute (USA) based on the 24-Form Simplified Tai Chi (Mendoza-Núñez et al., 2018). As a simplified version, the Eight-Form Tai Chi is characterized by concise content and simple movements. It eliminates strenuous actions such as breath-holding and sudden force exertion, making it easy to learn and memorize. The movements of the Eight-Form Tai Chi include the following: (1) Commencing Form (stepping out with the left foot to shoulder width, raising arms forward, bending knees, and pressing palms down); (2) Repulse Monkey; (3) Brush Knee and Twist Step on Both Side; (4) Wild Horse’s Mane; (5) Wave-Hand in Cloud; (6) Stand on one leg both side; (7) Heel Kick; (8) Grasp the Bird’s Tail; (9) Cross Hands; (10) Closing Form (flipping palms and separating hands, lowering arms, and bringing feet together). The movement chart for the Eight-Form Tai Chi is presented in **Figure 3**.

The Tai Chi intervention was delivered by professional martial arts instructors who conducted both the teaching and practice sessions. The intervention lasted for three months, with three 60-minute sessions per week. The first two weeks were designated as the instructional phase, adopting a “teaching-while-practicing” approach. During this period, two new movements were taught in each session (excluding the Commencing and Closing Forms). The structure of each session included a 10-minute warm-up, 20 minutes of learning new movements, 20 minutes of repetitive practice, and a 10-minute cool-down. Following the completion of instruction after two weeks, the study entered the practice phase. During this phase, the session structure was adjusted to a 5-minute warm-up, 50 minutes of continuous Tai Chi practice, and a 5-minute cool-down.

### 2.3 Statistical analysis

Upon completion of data collection, a database was established using Microsoft Excel 2016. Data compilation was performed using MATLAB R2021b, and statistical analyses were conducted using SPSS 26.0. Baseline characteristics, such as age, gender, and education level, were analyzed as follows: Categorical data were expressed as frequencies and percentages (%) and analyzed using Fisher’s exact test. For continuous variables that did not conform to a normal distribution, data were described using quartiles (median and interquartile range [IQR]) and analyzed using the Mann–Whitney U test. All outcome measures collected from both groups before and after the intervention are described as the mean ± standard deviation. Repeated measures analysis of variance (ANOVA) was used to analyze the changes in indicators between the two groups pre- and post-intervention. For repeated measures ANOVA, effect sizes were reported as partial eta squared (η²p). For *post-hoc* pairwise comparisons involving repeated measures, Cohen’s dz was calculated for significant differences. Cohen’s d was used for between-group comparisons of change scores. The primary outcomes of this study were gait spatiotemporal parameters. Secondary outcomes included lower limb joint angles at specific gait events and range of motion. The significance level for all statistical tests was set at α= 0.05, and p< 0.05 was considered statistically significant.

## 3 Results

### 3.1 Baseline characteristics

There were no statistically significant differences in the baseline characteristics between the two groups (p > 0.05). The detailed results are presented in **Table 1**.

**Table 1:** Baseline characteristics of participants (n=34).

Measure		Participants	Intervention	Control	Z	*P*
		(n=34)	(n=17)	(n=17)		
		n(%)	n(%)	n(%)		
Age	60∼69	25(73.5)	14(82.4)	11(64.7)	–	0.438
	70∼79	9(26.5)	3(17.6)	6(35.3)		
Gender	Female	25(73.5)	13(76.5)	12(70.6)	–	1.000
	Male	9(26.5)	4(23.5)	5(29.4)		
Education	<6years	9(26.5)	3(17.6)	6(35.3)	–	0.249
	6∼12years	20(58.8)	10(58.8)	10(58.8)		
	>12years	5(14.7)	4(23.5)	1(5.9)		
Hypertension	Yes	15(44.1)	6(35.3)	9(52.9)	–	0.491
	No	19(55.9)	11(64.7)	8(47.1)		
Hyperlipidemia	Yes	4(11.8)	1(5.9)	3(17.6)	–	0.601
	No	30(88.2)	16(94.1)	14(82.4)		
Exercise	0 sessions/week	6(17.6)	1(5.9)	5(29.4)	–	0.360
	1–3 sessions/week	3(8.8)	2(11.8)	1(5.9)		
	>3 sessions/week	25(73.5)	14(82.4)	11(64.7)		
MoCA(*M*(*Q1,Q3*))		23.0(21.0,24.5)	23.0(22.0,25.0)	23.0(19.0,24.0)	-1.731	0.092

Fisher’s exact test; M, median; Q1, lower quartile (25th percentile); Q3, upper quartile (75th percentile).

### 3.2 Results of gait temporal parameters in older adults with mci before and after Tai Chi intervention

Statistical analyses were performed on gait temporal parameters, including cadence, step time, gait cycle duration, stance phase percentage, and swing phase percentage, across different groups and tasks before and after the intervention. A two-way repeated measures analysis of variance (ANOVA) was conducted, with age and gender included as covariates. The results are summarized in **Table 2**. Regarding the changes in cadence pre- and post-intervention, the main effect of task was significant (p = 0.010, η²p = 0.28). Significant differences were observed in cadence changes among the three dual-task conditions: walking while counting, walking while carrying water, and walking while naming (p < 0.05). Regarding the change in the percentage of the right leg stance phase, the main effect of group was significant (p = 0.018, Cohen’s d = 0.84). In the experimental group, the percentage of the right leg stance phase increased across all three dual-task conditions post-intervention compared to baseline; however, the differences in changes between tasks were not statistically significant (p > 0.05). For changes in the remaining parameters, neither the main effects of time nor group were significant (p > 0.05).

**Table 2:** Changes in temporal gait parameters pre- and post-intervention: effects of group and task.

Measure			W-C	W-W	W-N	Task		Group		Task*Group	
						F	*P*	F	*P*	F	*P*
SF(steps/min)		Intervention	0.67 ± 10.81	-2.79 ± 8.28	-0.53 ± 9.20	4.987	**0.010**	0.193	0.664	1.061	0.353
		Control	0.26 ± 6.27	-1.87 ± 4.09	-2.17 ± 6.10						
GC (s)	L	Intervention	-0.05 ± 0.13	-0.05 ± 0.09	-0.02 ± 0.11	0.221	0.694	0.566	0.458	0.64	0.462
		Control	0.06 ± 0.29	0.03 ± 0.09	0.00 ± 0.05						
	R	Intervention	-0.05 ± 0.18	-0.02 ± 0.12	-0.01 ± 0.10	2.478	0.092	0.969	0.333	0.962	0.370
		Control	0.04 ± 0.12	0.01 ± 0.06	0.02 ± 0.15						
ST (s)	L	Intervention	-0.01 ± 0.13	0.02 ± 0.04	0.01 ± 0.06	2.621	0.108	0.005	0.942	0.918	0.364
		Control	-0.00 ± 0.10	-0.00 ± 0.03	0.02 ± 0.05						
	R	Intervention	-0.03 ± 0.10	-0.06 ± 0.38	0.00 ± 0.08	0.697	0.446	0.691	0.412	0.043	0.896
		Control	0.03 ± 0.09	-0.02 ± 0.07	0.02 ± 0.08						
Stance Phase(%)	L	Intervention	0.36 ± 2.58	0.77 ± 1.40	1.27 ± 2.20	0.821	0.445	0.714	0.405	0.258	0.774
		Control	-0.04 ± 2.71	0.26 ± 3.44	0.23 ± 2.19						
	R	Intervention	1.05 ± 6.75	1.49 ± 2.27	1.35 ± 6.59	3.055	0.082	6.309	**0.018**	0.157	0.744
		Control	-0.29 ± 2.94	0.27 ± 1.11	0.38 ± 2.28						
Swing Phase (%)	L	Intervention	-0.36 ± 2.58	-0.77 ± 1.40	-1.27 ± 2.20	1.181	0.314	0.72	0.403	0.112	0.894
		Control	0.34 ± 2.92	-0.33 ± 3.41	-0.34 ± 2.12						
	R	Intervention	-2.31 ± 5.80	-1.51 ± 2.30	0.69 ± 4.95	0.297	0.674	3.142	0.086	1.551	0.225
		Control	0.32 ± 2.96	-0.27 ± 1.11	0.69 ± 4.95						

W-C, walking-counting dual-task; W-W, walking-water carrying dual-task; W-N, walking-naming dual-task; L, left; R, right.

Bold value indicates significant differences (p < 0.05).

Multiple comparisons were conducted to analyze the differences in cadence changes across the three dual-task conditions: walking while counting, walking while carrying water, and walking while naming. The results are presented in **Table 3**. A statistically significant difference was observed in cadence changes between the walking-while-carrying-water task and the walking-while-naming task(p = 0.027, Cohen’s dz = 0.52). No statistically significant differences were found between the other dual-task conditions (p > 0.05).

**Table 3:** Comparison of stride frequency variations across different tasks.

Task1	Task2	*P*
W-C	W–W	0.238
W–W	W–N	**0.027**
W–N	W-C	1.000

W-C, walking-counting dual-task; W-W, walking-water carrying dual-task; W-N, walking-naming dual-task.

Bold value indicates significant differences (p < 0.05).

The asterisk (*) represents "×".

### 3.3 Results of gait spatial parameters in older adults with MCI before and after Tai Chi intervention

Statistical analyses were performed on the changes in general gait spatial parameters, including step length, stride length, and step width-across different groups and tasks before and after the intervention. The detailed results are presented in **Table 4**. Regarding the changes in normalized step length, stride length, and step width pre- and post-intervention, the main effects of group were significant (step length: p = 0.039, Cohen’s d = 0.78; stride length: p = 0.045, Cohen’s d = 0.75; step width: p = 0.002, Cohen’s d = 1.42). In the experimental group, both step length and stride length increased across all three dual-task conditions post-intervention compared to baseline, while step width decreased. However, the differences in changes between tasks were not statistically significant, and no significant task-by-group interaction effects were observed (p > 0.05).

**Table 4:** Analysis of intervention effects and group × task interactions on normalized step length, stride length, and step width.

Measure		W-C	W–W	W–N	Task		Group		Task*Group	
					F	*P*	F	*P*	F	*p*
SL (%)	Intervention	2.81 ± 2.52	1.76 ± 6.52	2.74 ± 3.86	0.823	0.445	**4.766**	**0.039**	0.681	0.511
	Control	-0.46 ± 2.06	0.07 ± 2.00	-0.36 ± 2.36						
SL_2_ (%)	Intervention	2.96 ± 5.19	1.82 ± 6.08	2.83 ± 4.79	0.020	0.98	**4.454**	**0.045**	1.271	0.289
	Control	-0.88 ± 4.27	0.28 ± 2.80	-1.03 ± 3.96						
SW (cm)	Intervention	-1.84 ± 0.94	-1.92 ± 2.50	-1.87 ± 2.01	0.194	0.826	**25.036**	**0.002**	0.391	0.684
	Control	1.10 ± 1.34	0.36 ± 1.14	0.18 ± 0.97						

W-C, walking-counting dual-task; W-W, walking-water carrying dual-task; W-N, walking-naming dual-task; SL2, Stride length.

Bold value indicates significant differences (p < 0.05).

The asterisk (*) represents "×".

The sagittal plane joint angle parameters of the hip, knee, and ankle were measured for the different groups and tasks before and after the intervention. The detailed results are presented in **Table 5**.

**Table 5:** Changes in hip joint kinematics pre- and post-intervention: effects of group and task.

Measure		W–C	W–W	W–N	Task		Group		Task*Group	
					F	*P*	F	*P*	F	*P*
Right Foot Initial Contact	Intervention	0.70 ± 8.00	-0.63 ± 5.53	0.16 ± 8.81	0.445	0.619	0.041	0.841	0.682	0.493
	Control	0.29 ± 2.30	0.55 ± 1.51	-0.41 ± 1.31						
Left Foot Toe-Off	Intervention	-1.51 ± 8.19	-3.51 ± 6.16	-2.84 ± 7.10	1.730	0.191	2.295	0.140	1.131	0.324
	Control	0.89 ± 4.19	0.80 ± 2.96	-0.86 ± 2.13						
Left Foot Initial Contact	Intervention	-7.05 ± 8.64	-5.27 ± 5.09	-6.54 ± 6.74	0.712	0.495	18.051	**<0.001**	2.620	0.081
	Control	0.23 ± 1.90	-0.73 ± 1.94	1.60 ± 3.31						
Right Foot Toe-Off	Intervention	-3.36 ± 6.06	-2.30 ± 6.27	-3.21 ± 5.89	1.629	0.205	3.786	0.061	0.289	0.750
	Control	-0.43 ± 3.96	0.19 ± 1.58	0.09 ± 2.13						
Subsequent Right Foot Contact	Intervention	1.12 ± 8.00	-0.57 ± 5.72	-0.05 ± 9.16	0.766	0.466	0.290	0.594	0.538	0.581
	Control	0.08 ± 1.77	-0.25 ± 1.10	-0.97 ± 1.67						
Peak Flexion	Intervention	-0.60 ± 5.97	-0.21 ± 5.51	-0.96 ± 7.28	0.494	0.613	0.009	0.924	0.245	0.784
	Control	0.76 ± 1.56	0.05 ± 1.11	-0.55 ± 1.40						
Peak Extension	Intervention	-6.46 ± 6.11	-5.62 ± 5.36	-6.17 ± 5.89	2.067	0.136	**16.993**	**<0.001**	**4.870**	**0.011**
	Control	-0.50 ± 0.86	-0.80 ± 1.48	1.04 ± 2.11						
Range of Motion	Intervention	5.86 ± 3.31	5.41 ± 3.88	5.20 ± 3.21	2.652	0.079	**61.047**	**<0.001**	2.985	0.058
	Control	0.75 ± 1.76	0.84 ± 2.11	-1.80 ± 2.45						

W-C, walking-counting dual-task; W-W, walking-water carrying dual-task; W-N: walking-naming dual-task.

Bold value indicates significant differences (p < 0.05).

The asterisk (*) represents "×".

Regarding hip joint angle characteristics, as shown in **Table 5**, the main effects of group were significant for the changes in hip angle at the moment of left foot contact (p < 0.001, Cohen’s d = 1.56), maximum hip extension (p < 0.001, Cohen’s d = 1.63), and flexion-extension range of motion (ROM) (p < 0.001, Cohen’s d = 2.12). Specifically, the hip ROM increased in the experimental group. A significant task-by-group interaction effect was observed for the change in maximum hip extension (p = 0.011, η²p = 0.24), whereas no interaction effects were found for the other parameters (p > 0.05). A simple effects analysis was conducted on the change in maximum hip extension, where the interaction effect was significant. The results, shown in **Table 6**, indicated statistically significant differences in the change in maximum hip extension between the two groups across all tasks (W-C: p = 0.001, Cohen’s d = 1.49; W-W: p = 0.002, Cohen’s d = 1.37; W-N: p < 0.001, Cohen’s d = 1.58). Regarding the differences between tasks, significant differences in change values were observed in the control group between the walking-while-carrying-water and walking-while-naming tasks (p = 0.019, Cohen’s dz = 0.58), as well as between the walking-while-counting and walking-while-naming tasks (p = 0.019, Cohen’s dz = 0.58).

**Table 6:** Simple effects analysis on the change in peak hip extension angle.

Comparison type	Condition 1	Condition 1	F	*P*
Between-Groups Comparison (Intervention vs. Control)	W-C	–	1.493	**0.001**
	W–W	–	1.366	**0.002**
	W–N	–	1.578	**<0.001**
Between-Tasks Comparison (Intervention)	W-C	W–W	1.545	0.230
	W–W	W–N	–	–
	W–N	W-C	–	–
Between-Tasks Comparison (Control)	W-C	W–W	4.554	0.019*
	W–W	W–N	–	–
	W–N	W-C	–	–

W-C, walking-counting dual-task; W-W, walking-water carrying dual-task; W-N, walking-naming dual-task; *:*p* <.05 for the difference in the change of peak hip extension angle between two dual-task conditions.

Bold value indicates significant differences (p < 0.05).

Regarding knee joint angle characteristics, as indicated in **Table 7**, the main effect of group was significant for the change in knee flexion angle at the moment of left foot contact (p < 0.001, Cohen’s d = 1.38). Regarding the change in knee flexion angle at the moment of right foot recontact, the main effect of task was significant (p = 0.044, η²p = 0.18). Multiple comparisons were conducted for the tasks, with the results presented in **Table 8**. A significant difference was observed in the change in knee flexion angle between the walking-while-carrying-water and walking-while-naming tasks (p = 0.038, Cohen’s dz = 0.48).

**Table 7:** Analysis of knee joint angle changes: effects of intervention, group, and task.

Measure		W-C	W–W	W–N	Task		Group		Task*Group	
					*F*	*P*	*F*	*P*	*F*	*P*
Right Foot Initial Contact	Intervention	2.10 ± 7.19	2.23 ± 6.35	1.09 ± 6.67	1.372	0.262	0.728	0.400	0.092	0.912
	Control	0.31 ± 2.81	0.75 ± 1.39	-0.24 ± 0.93						
Left Foot Toe-Off	Intervention	-0.25 ± 6.30	-0.87 ± 5.35	-1.76 ± 6.51	1.125	0.327	0.792	0.381	0.421	0.637
	Control	0.72 ± 2.91	2.15 ± 8.87	-1.38 ± 2.65						
Left Foot Initial Contact	Intervention	-5.53 ± 5.30	-6.33 ± 6.36	-5.75 ± 6.46	0.026	0.974	15.481	**<0.001**	0.523	0.595
	Control	1.30 ± 4.30	-0.81 ± 2.23	0.70 ± 3.11						
Right Foot Toe-Off	Intervention	-1.72 ± 8.85	-3.15 ± 8.26	-2.00 ± 9.55	0.395	0.675	0.522	0.476	0.18	0.836
	Control	-0.91 ± 4.28	-1.17 ± 7.97	-0.59 ± 2.89						
Subsequent Right Foot Contact​	Intervention	1.93 ± 6.45	1.96 ± 7.54	0.64 ± 7.43	3.284	**0.044**	0.859	0.361	0.214	0.808
	Control	0.44 ± 2.36	0.18 ± 1.82	-1.17 ± 2.16						
Peak Flexion​	Intervention	-1.65 ± 6.90	-2.43 ± 7.18	-2.42 ± 8.54	0.342	0.684	1.026	0.365	1.038	0.316
	Control	-0.70 ± 4.04	0.47 ± 1.32	-0.36 ± 3.15						
Peak Extension​	Intervention	0.94 ± 6.46	1.56 ± 7.03	0.33 ± 7.37	2.701	0.075	1.162	0.32	0.135	0.716
	Control	0.32 ± 1.48	0.48 ± 2.20	0.48 ± 2.11						
Range of Motion	Intervention	-2.58 ± 8.31	-3.99 ± 6.59	-2.76 ± 7.76	1.833	0.175	1.709	0.19	1.782	0.192
	Control	-1.02 ± 4.17	0.00 ± 2.56	-0.84 ± 4.23						

W-C, walking-counting dual-task; W-W, walking-water carrying dual-task; W-N, walking-naming dual-task.

Bold value indicates significant differences (p < 0.05).

The asterisk (*) represents "×".

**Table 8:** Comparison of knee joint kinematics at subsequent right foot contact: a task-wise analysis.

Task1	Task2	*P*
W-C	W-W	1.000
W-W	W-N	**0.038**
W-N	W-C	0.051

W-C, walking-counting dual-task; W-W, walking-water carrying dual-task; W-N, walking-naming dual-task.

Bold value indicates significant differences (p < 0.05).

Regarding ankle joint angle characteristics, as indicated in **Table 9**, the main effect of group was significant for the change in ankle dorsiflexion angle at the moment of right foot toe-off (p = 0.003, Cohen’s d = 1.05). Regarding the change in ankle dorsiflexion angle at the moment of left foot contact, the main effect of task was significant (p = 0.036, η²p = 0.19). Multiple comparisons were performed for the tasks, and the results are presented in **Table 10**. The analysis revealed no statistically significant differences in the change in ankle angle at the moment of left foot contact between any pair of dual-task conditions (p > 0.05).

**Table 9:** Analysis of ankle joint angle changes: effects of intervention, group, and task.

Measure		W-C	W–W	W–N	Task		Group		Task*Group	
					F	*P*	F	*P*	F	*P*
Right Foot Initial Contact	Intervention	-1.1 ± 4.48	0.13 ± 4.23	-0.97 ± 5.64	0.211	0.81	0.51	0.481	0.067	0.935
	Control	-0.44 ± 3.59	0.65 ± 3.08	0.25 ± 2.68						
Left Foot Toe-Off	Intervention	-0.14 ± 4.32	0.05 ± 3.98	-0.54 ± 4.46	1.492	0.232	0.012	0.913	0.286	0.753
	Control	-0.56 ± 2.73	-0.57 ± 1.59	-0.34 ± 1.94						
Left Foot Initial Contact	Intervention	1.06 ± 6.48	-0.06 ± 4.80	-0.51 ± 5.43	3.515	**0.036**	0.507	0.482	0.601	0.551
	Control	-0.21 ± 2.60	-0.23 ± 1.51	-1.10 ± 2.95						
Right Foot Toe-Off	Intervention	-3.61 ± 6.57	-3.61 ± 6.92	-5.15 ± 5.13	0.851	0.432	10.617	**0.003**	3.073	0.054
	Control	0.81 ± 2.91	-1.34 ± 3.21	1.79 ± 3.17						
Subsequent Right Foot Contact	Intervention	-0.58 ± 4.80	-0.55 ± 4.12	-0.72 ± 0.00	0.234	0.792	0.006	0.937	0.262	0.771
	Control	-0.90 ± 2.89	-0.86 ± 2.86	0.02 ± 2.85						
Peak Dorsiflexion	Intervention	2.12 ± 7.26	1.69 ± 5.52	1.62 ± 7.27	1.386	0.258	1.858	0.183	0.195	0.801
	Control	0.40 ± 1.47	0.12 ± 1.70	-0.35 ± 1.91						
Peak Plantarflexion	Intervention	-2.30 ± 3.85	-1.82 ± 6.12	-3.29 ± 6.02	1.976	0.148	0.928	0.401	1.409	0.245
	Control	-1.42 ± 2.66	-1.40 ± 3.07	-0.31 ± 1.95						
Range of Motion	Intervention	4.41 ± 6.97	4.51 ± 8.28	3.55 ± 6.05	0.443	0.630	3.399	0.075	0.138	0.713
	Control	0.82 ± 3.11	1.52 ± 3.30	-0.04 ± 2.52						

W-C, walking-counting dual-task; W-W, walking-water carrying dual-task; W-N, walking-naming dual-task.

Bold value indicates significant differences (p < 0.05).

The asterisk (*) represents "×".

**Table 10:** Comparison of ankle joint kinematics at left foot contact: a task-wise analysis.

Task1	Task2	*P*
W-C	W-W	1.000
W-W	W-N	0.846
W-N	W-C	0.396

W-C, walking-counting dual-task; W-W, walking-water carrying dual-task; W-N, walking-naming dual-task.

### 3.4 Results of cognitive performance parameters in older adults with MCI before and after Tai Chi intervention

The statistical analysis results of cognitive performance are presented in **Tables 11**, **12**. The Tai Chi group significantly improved MoCA score post-intervention (28.0 [27.0–29.5], p < 0.001), while the control group showed no change (23.0 [18.5–23.0], p = 0.794). Between-group comparison revealed significantly higher scores in the Tai Chi group after the intervention (p < 0.001). For dual-task performance, AR and CNR showed no significant main effects of task or group, nor interactions (all p > 0.05). For RT, significant main effects were found for task (F = 14.612, p = 0.001, η²p = 0.32) and group (F = 5.384, p = 0.030, Cohen’s d = 0.78).

**Table 11:** Analysis of the impact of interventions on cognitive performance before and after.

Measure		Single-task	Dual-task	Task		Group		Task*Group	
				F	*P*	F	*P*	F	*P*
AR (%)	Intervention	0.89 ± 7.16	1.59 ± 10.33	0.0453	0.508	0.214	0.649	0.025	0.876
	Control	1.59 ± 10.33	1.48 ± 16.40						
RT (ms)	Intervention	–130.93 ± 227.6	–107.33 ± 186.5	**14.612**	**0.001**	**5.384**	**0.030**	0.101	0.754
	Control	105.99 ± 387.1	–8.44 ± 31.0						
CNR	Intervention	0.42 ± 2.15	0.38 ± 2.08	0.214	0.647	0.156	0.695	0.089	0.768
	Control	0.21 ± 1.98	0.15 ± 2.11						

NAR, accuracy rates; RT, reaction times; CNR, number of correct naming responses.

Bold value indicates significant differences (p < 0.05).

The asterisk (*) represents "×".

**Table 12:** Simple effects analysis on the change in RT.

Group	F	*P*
Intervention	5.626	**0.027**
Control	1.275	0.271

Bold value indicates significant differences (p < 0.05).

## 4 Discussion

### 4.1 Effects of 8-style Tai Chi exercise on walking posture

Older adults with MCI typically exhibit characteristics such as gait instability, reduced cadence, and increased step time; these changes are closely correlated with cognitive decline (Hackney and Earhart, 2008). The results of this study indicate that 8-style Tai Chi training significantly increased the proportion of the right stance phase during dual-task walking in the elderly MCI population. An increase in the proportion of the stance phase generally suggests an improvement in gait stability. Zhang et al. (2006) found that Tai Chi training could significantly increase the stance phase proportion during walking in older adults, a finding consistent with the results of the present study. Furthermore, the results showed a significant between-group difference in the change in the right stance phase proportion, whereas the difference for the left foot was not significant. This discrepancy may be attributed to potential baseline lower limb incoordination among the subjects. Additionally, the increased cognitive load during dual-task performance may compromise neuromuscular control in older adults with MCI, resulting in a greater reliance on the right leg, typically the dominant limb, than on the left leg. The lack of statistical significance in other parameters may be due to the relatively short duration of the intervention in this study, which may have been insufficient to elicit significant improvements across all gait parameters.

In addition, the spatial parameters of gait in older adults with MCI are typically characterized by shortened step length, reduced stride length, and increased step width. Kuan et al. (2021) investigated the gait characteristics of middle-aged and elderly community residents with mild cognitive impairment. They found that the MCI population exhibited poorer performance in both spatial (stride length, step height, step width) and temporal (gait speed, stride velocity, swing velocity) domains compared to healthy controls. Specifically, their stride length, step height, gait speed, and stride velocity were lower. The results of the present study indicate that 8-style Tai Chi training significantly increased step length and stride length while decreasing step width during dual-task walking in older adults with MCI. Step length and stride length are critical parameters reflecting gait efficiency. Previous studies have demonstrated that 3 months of Tai Chi training can effectively increase step length and stride length in older adults (Li et al., 2012), a finding consistent with the results of this study. Zhang et al. (2006) suggested that Tai Chi training increases step length by improving movement coordination and reducing energy expenditure during gait. Meanwhile, Li et al. (2004) proposed that Tai Chi training increases step length by enhancing lower limb muscle strength, thereby improving propulsive force during walking. Step width is a key parameter reflecting gait stability; it is generally believed that a wider step width correlates with greater stability. However, Chen et al. (2024) found that long-term Tai Chi practice effectively increased step width in elderly women. This contradicts the findings of the present study. This discrepancy may be attributed to the specific target population of this study (older adults with MCI). Due to decreased postural control, this population may spontaneously increase step width during daily walking as a compensatory strategy to maintain balance, driven by caution and a fear of falling. Following Tai Chi training, their cognitive and postural control abilities improved, reducing the need for this compensatory widening to maintain stability. Consequently, a decrease in step width was observed postintervention.

During walking, flexion and extension movements of the hip, knee, and ankle joints are the most prominent. The angular changes in the lower limb joints in the sagittal plane are critical parameters in gait analysis. The findings of this study indicate that 8-style Tai Chi training effectively improves hip joint mobility in older adults with MCI. With advancing age, the joint range of motion in older adults typically decreases. This is primarily characterized by reduced hip extension, increased knee flexion, and increased ankle dorsiflexion. The results demonstrate that the peak hip extension and the total flexion-extension ROM significantly increased in the MCI group during walking following the intervention. Tai Chi footwork requires practitioners to rotate the hips moving upward, laterally, and downward to facilitate the transition of the center of gravity forward and backward. This practice increases the frequency of hip joint activation and significantly enhances the vertical amplitude of hip movement (Pan et al., 2025). Furthermore, the ‘Bow Stance’ (Gongbu) requires full extension of the hip and knee of the trailing leg, which further increases the range of motion of the hip joint. Consequently, tai chi training is effective in improving hip joint flexibility. Wehner et al. (2021) found that Tai Chi practice fully engages the major joints of the lower limbs and the spine, playing a positive role in enhancing joint mobility, flexibility, and range of motion. This finding is consistent with the results of the present study.

### 4.2 Effects of different task conditions on gait in older adults with MCI pre- and post-8-style Tai Chi training

The results of this study indicate significant differences in cadence changes between the walking-while-holding-water dual-task condition and the walking-while-naming dual-task condition. The water-holding task, classified as a motor-execution dual task, primarily activates the prefrontal-cerebellar motor coordination network (Koziol et al., 2012). It requires subjects to achieve precise postural control while simultaneously maintaining gait stability. In contrast, the naming task, classified as a language-processing dual task, primarily involves the language processing regions of the left inferior frontal gyrus and the temporal lobe. This disparity in neural resource competition patterns resulted in a more significant reduction in cadence among MCI patients during the naming task. This phenomenon may be associated with altered resource allocation strategies within the central executive system (Persad et al., 2008). Motor-cognitive dual tasks involve information processing across different modalities, thereby demanding greater attentional resources and task-switching abilities. Conversely, motor-motor dual tasks primarily involve coordination within the same modality, resulting in a lower dependency on cognitive resources.

Furthermore, the results of this study indicate significant differences within the control group regarding the change in peak hip extension between the walking-while-holding-water condition and the walking-while-naming condition. Additionally, the change observed under the walking-while-naming condition differed significantly from that under the walking-while-counting condition. When processing dual tasks involving object manipulation, healthy older adults preferentially utilize the basal ganglia-thalamocortical circuits for automated motor regulation (Wong et al., 2015). Consequently, the kinematic changes in the hip joint induced by the water-holding task are relatively minor. In contrast, patients with MCI experience a reduction in striatal dopaminergic neurotransmitters, leading to impaired automaticity of motor programs. This deficit necessitates a greater reliance on the prefrontal cortex for conscious (executive) control. This compensatory mechanism is particularly pronounced during counting tasks, which require the active engagement of working memory (Wu and Hallett, 2005).

Data regarding the knee flexion angle at the moment of right foot ground contact (initial contact) revealed a significant difference between the water-holding and naming tasks. This disparity may be attributed to the water-holding task’s requirement for continuous visual-proprioceptive integration, which prompts an increased knee flexion angle during the landing phase to enhance shock absorption capabilities. Conversely, the semantic retrieval demands elicited by the naming task may affect knee joint control through two potential mechanisms: (1) causing a delay in motor program initiation via hyperactivation of the anterior cingulate cortex-supplementary motor area (ACC-SMA) circuit or (2) disrupting the efficiency of proprioceptive integration through aberrant activity in the hippocampal-thalamic pathway. Consistent with prior research, there are significant differences in the impact of motor-cognitive dual tasks versus motor-motor dual tasks on gait function in older adults with MCI (Wu and Hallett, 2005). The present study demonstrates that motor-cognitive dual tasks generally exert a greater negative impact on gait function than motor-motor dual tasks. This is likely because motor-cognitive tasks demand substantial cognitive resources; given that cognitive function is already compromised in older adults with MCI, they struggle to allocate attentional resources effectively. In contrast, motor-motor dual tasks primarily involve motor coordination and balance control, thereby placing relatively lower demands on cognitive resources.

### 4.3 Limitations

The final number of participants who completed the intervention in this study met the minimum sample size requirement. Although the control group received bi-weekly follow-ups to balance attention across groups, the absence of an active control condition (e.g., stretching or health education) means we cannot entirely rule out the influence of non-specific factors such as increased social interaction or expectation. Future studies incorporating active control groups would help further isolate the specific effects of Tai Chi. Furthermore, this study was limited to a comparison of outcomes before and after a three-month intervention period and did not conduct a comparative analysis of different intervention durations. Future research should investigate the effects of 8-style Tai Chi training at varying durations and frequencies to determine the optimal exercise protocol for older adults with MCI. Fourth, given the exploratory nature of this study and the numerous gait parameters analyzed, we did not apply strict multiple comparison corrections, as such adjustments could increase type II error and obscure potentially meaningful effects. Instead, we reported effect sizes alongside p-values to facilitate interpretation of the observed effects. Nevertheless, the risk of type I error remains, and our findings should be considered hypothesis-generating rather than confirmatory. Future studies with larger sample sizes are needed to validate these results.

## 5 Conclusions

A three-month, supervised 8-form Tai Chi intervention (3 sessions/week, 60 min/session) significantly improved dual-task gait stability, balance, and overall gait performance in older adults with mild cognitive impairment, thereby effectively reducing fall risk. Notably, enhancements were more pronounced under the walking-counting dual-task condition, suggesting that Tai Chi may optimize postural control by enhancing cognitive-motor integration and attentional resource allocation under competing task demands. This study provides robust evidence supporting Tai Chi as a safe, cost-effective, and scalable non-pharmacological intervention for fall prevention in MCI patients. Integrating this evidence-based practice into public health strategies could alleviate fall-related socioeconomic burdens and enhance quality of life, contributing substantially to healthy aging initiatives.

## Data Availability

The raw data supporting the conclusions of this article will be made available by the authors, without undue reservation.
